# What can you do with 0.1× genome coverage? A case study based on a genome survey of the scuttle fly *Megaselia scalaris *(Phoridae)

**DOI:** 10.1186/1471-2164-10-382

**Published:** 2009-08-18

**Authors:** David A Rasmussen, Mohamed AF Noor

**Affiliations:** 1 Department of Biology, Duke University, Durham, NC 27708 USA

## Abstract

**Background:**

The declining cost of DNA sequencing is making genome sequencing a feasible option for more organisms, including many of interest to ecologists and evolutionary biologists. While obtaining high-depth, completely assembled genome sequences for most non-model organisms remains challenging, low-coverage genome survey sequences (GSS) can provide a wealth of biologically useful information at low cost. Here, using a random pyrosequencing approach, we sequence the genome of the scuttle fly *Megaselia scalaris *and evaluate the utility of our low-coverage GSS approach.

**Results:**

Random pyrosequencing of the *M. scalaris *genome provided a depth of coverage (0.05-0.1x) much lower than typical GSS studies. We demonstrate that, even with extremely low-coverage sequencing, bioinformatics approaches can yield extensive information about functional and repetitive elements. We also use our GSS data to develop genomic resources such as a nearly complete mitochondrial genome sequence and microsatellite markers for *M. scalaris*.

**Conclusion:**

We conclude that low-coverage genome surveys are effective at generating useful information about organisms currently lacking genomic sequence data.

## Background

Next-generation sequencing technologies, such as the 454 (Roche Applied Science) and Solexa (Illumina) platforms, provide researchers working on emerging- and non-model species an affordable means of addressing a wide range of questions [1]. While completely assembled genomes of non-model organisms may not be easily obtained, very low-coverage shotgun sequencing can be used for various applications, such as to identify microsatellites for population genetic analyses [2,3]. Low coverage genome survey sequences (GSS) can also provide information about gene content, polymorphisms, functional elements and repetitive elements [4].

In support of the utility of low-coverage sequencing, simulations have shown that most of the coding sequence in a genome can be surveyed with less than 2× genomic coverage [5]. As a case in point, the original 1.5× assembly of the dog genome provided partial sequence of thousands of dog orthologs of human genes [6]. At even lower coverage, Wernersson et al. [7] were able to recover 38% of the coding fraction of the mouse-human alignment with only 0.66× coverage of the pig genome. Furthermore, low-coverage survey sequencing appears to be an efficient way of identifying common repetitive DNA sequences. For example, a large fraction of the repetitive DNA sequences in the complex, highly repetitive barley genome of were computationally identified with only ~10% of the genome sequenced [8]. These studies suggest that as next generation sequencing becomes more widespread, low-coverage genome surveys will play a prominent role in studies of non-model species.

To gauge the suitability of genome surveys at depths of coverage lower than those used in previous studies, we partially sequenced the genome of the scuttle fly *Megaselia scalaris *(Phoridae) by 454 pyrosequencing. While no genomic resources had been developed for this species prior to this point, *M. scalaris *has a rich history in biological research [reviewed in [9]] and can serve as a representative of a potentially emerging model species for ecology and evolutionary biology. To this end, one-fourth of a standard 454 run was conducted with randomly sheared genomic DNA. Based on flow cytometry estimates of genome size, the depth of coverage across the genome was approximately 0.05-0.1x. However, our low-coverage likely reflects the position of many researchers using next-generation technologies for sequencing in non-model eukaryotes with large, complex genomes. We therefore view our low-coverage as a bioinformatics challenge and focus on analyses that can be conducted with limited GSS data.

Even with only ~0.1× coverage, we were able to generate a considerable amount of biologically useful information and genomic resources for *M. scalaris*. First, because of the substantial impact repetitive elements (REs) can have on the structure and evolution of genomes [10,11], we searched for REs in *M. scalaris *based on homology to well-characterized REs. Novel or lineage-specific tandem REs were then identified by using a custom program we developed. The effectiveness of these computational procedures was evaluated by performing the same analyses on low-coverage sequence simultaneously generated from *Drosophila pseudoobscura*, for which a well-annotated reference genome sequence is available [12]. Secondly, we assembled a nearly complete sequence for the ~15.4 kb mitochondrial (mt) genome of *M. scalaris*. Thirdly, we looked for microsatellite loci to develop molecular markers for *M. scalaris*. Finally, we identified coding regions and other functional elements in the *M. scalaris *genome by comparisons to the completed genomes of *Drosophila melanogaster *and other Dipterans. A discussion of the utility and concerns raised by extremely low-coverage survey sequencing is also presented.

## Results

A total of 129,080 sequence reads with a mean read length of 231 bp were generated from 454 sequencing of randomly sheared *Megaselia scalaris *genomic DNA. If we assume the genome size to be approximately 540 megabases, as estimated from our flow cytometry, this coverage amounts to 0.055×. If instead the genome size is 330 megabases [13], we have 0.090× coverage of the genome.

To expedite analysis, the full set of sequences was filtered to remove redundant reads that were shorter, internal fragments of longer reads. The non-redundant set contained 96,625 reads and is hereinafter referred to as the genome survey sequence (GSS) set. Table 1 summarizes the proportions of redundant, mitochondrial and identified coding and repetitive (see below) sequence reads contained in the GSS set. The nucleotide content of the GSS reads showed that the *Megaselia *genome is G-C poor (32%) compared to A-T content (68%). Removing repetitive and mitochondrial sequences from the GSS further increased the nucleotide usage bias to a G-C content of 30%.

**Table 1:** Number and percentage of different types of sequence reads in the *M. scalaris *Genome Sequence Survey set.

Sequence Type	Number of GSS Reads	Percentage
All Sequences Combined	129,080	100%
Non-Redundant (Filtered) Sequences	96.625	74.90%
Mitochondrial Sequences	648	0.50%
Predicted Coding Sequences*	520	0.40%
Predicted Repetitive Sequences*	105	0.08%

*Based on significant similarity to known elements in other Dipteran genomes

### Survey of Repetitive Elements (REs)

Two approaches were used to identify repetitive elements in the *M. scalaris *genome. The first approach relied on homology of repeats to well-described REs in publicly available databases. A total of 102 REs were identified on the basis of similarity to known elements in the *Drosophila *and *Anopheles *RepBase libraries (Additional file 1). Based on the number of reads with significant similarity to various classes of REs, retroelements appear more abundant in copy number than DNA transposons, as is the tendency in most eukaryotes [14]. LTR retroelements were the most abundant class (45%), with many copies of *gypsy *and *copia *elements identified. The second most abundant class was non-LTR elements (LINEs), representing 38% of identified REs, including many *jockey *elements. Only 4% of identified REs were DNA transposons.

The second approach used our REFinder.plx http://www.biology.duke.edu/noorlab/NoorSoft.html program to identify tandem REs *de novo*. This method has the advantage of detecting previously uncharacterized or highly divergent REs, but was not designed to detect interspersed elements. Before running the program on the *M. scalaris *GSS reads, we tested the program on genome survey sequences from *D. pseudoobscura bogotana*, which were generated from the same 454 run and at the same coverage as the *M. scalaris *data. This allowed us to optimize parameters in the REFinder.plx program and to test the program's ability to detect previously described REs in the *D. pseudoobscura *genome. The contigs generated by the program matched single units of elements known in *D. pseudoobscura *such as *Leviathan *[15], a *bilbo *non-LTR, and ITS sequences. Furthermore, to confirm that REFinder.plx correctly assembles repetitive sequences, contigs that the program generated were queried against the assembled *D. pseudoobscura *reference genome. In most cases, our contigs aligned to stretches of the *D. pseudoosbcura *genome that were as long or just slightly shorter than the contigs generated by the program. That the alignments spanned the length of multiple reads suggests that these contigs were correctly assembled rather than artificial hybrid sequences.

Starting from a set of 1,741 candidate repeat sequences that were present in at least two copies in the *M. scalaris *GSS reads, REFinder.plx identified 340 tandem repeats, 251 of which were unique repeats that did not show significant similarity to other computationally identified REs (Additional file 2). The average coverage across all unique repeat motifs, calculated from coverage across 40 bp windows, was 19.93 (SD = 33.96) with a few repeat motifs reaching as great as 200× coverage. This high level of redundancy in repeat coverage suggests that most of the identified REs are present in high copy number in the *M. scalaris *genome. Furthermore, for 239 of the identified REs, no significant hits were found by BLAST searches of GenBank, suggesting that most of the identified REs were unique to *M. scalaris *or previously uncharacterized in other species.

### The *M. scalaris *Mitochondrial Genome

**Figure 1:**
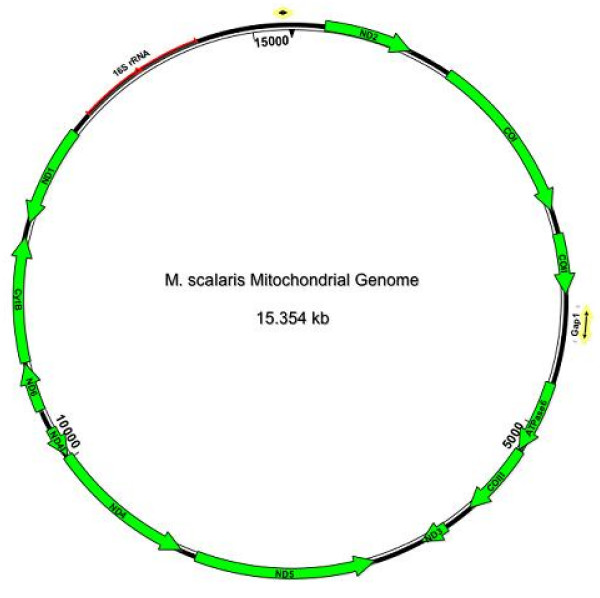
**Map of the *M. scalaris *Mitochondrial Genome**. Map of the *M. scalaris *mitochondrial genome showing the positions of the protein-coding genes (green arrows), 16S ribosomal RNAs (red line) and the gaps in our sequence (external yellow arrows).

We initially assembled the *M. scalaris *mitochondrial (mt) genome by aligning contiguous but overlapping sequence reads while using the *D. melanogaster *mt genome as a scaffold. From this initial assembly, 648 mitochondrial GSS sequences were identified and aligned into several large contigs. Gaps between contigs were amplified and sequenced by traditional Sanger sequencing when possible. There were two gaps for which we could not obtain high-quality sequence. The first is located between cytochrome c oxidase subunit II and ATPase subunit 6 and is flanked by A-T homopolymer tracts (Figure 1). Based on the size of a PCR fragment that spans this gap, it is approximately 300 bp long. The second gap is located between the 16S ribosomal RNAs and NADH dehyrdrogenase subunit 2, but we do not have an estimate for its length since it falls within a highly A-T rich region that varies in size among other Dipterans and we could not successfully amplify a fragment spanning the gap.

**Figure 2:**
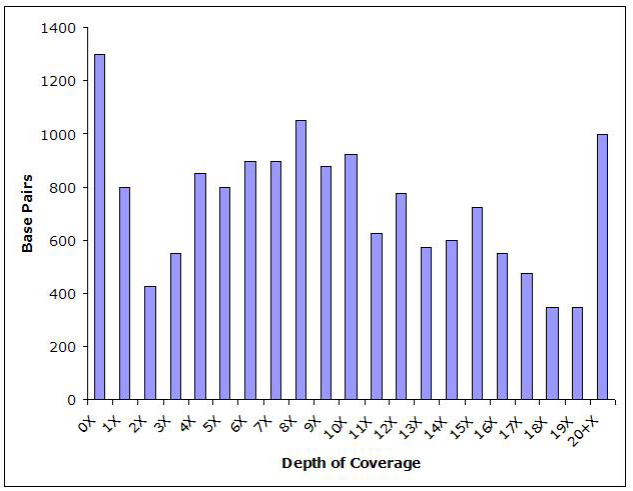
**Sequence Coverage Across the *M. scalaris *Mitochondrial Genome**. The depths of coverage, averaged across 25 bp windows, attained from genome survey sequences of the mitochondrial genome of *M. scalaris*. The vertical axis represents the number of nucleotide bases with the corresponding depth of coverage.

Although we did not obtain complete coverage, we did generate a 15.4 kb consensus sequence for the *M. scalaris *mt genome (Additional file 3). Coverage across the mt genome was high except in extremely A-T rich regions (the gaps mentioned above), where no 454 sequence traces were recovered. The mean depth of coverage was greater than 10×, with smaller regions attaining depths greater than 20× (Figure 2). Overall, the *M. scalaris *mt genome is similar to that of other Dipterans. The A-T content is high, at ~80%. The arrangement of protein-coding genes and the small and large ribosomal subunit RNAs is identical to that of most other arthropods (Boore, 1999), although ATPase subunit 8 was not present in our assembly (Figure 1). The most parsimonious explanation for the absence of ATPase subunit 8 is that the gene is located within Gap 1 of our assembly between cytochrome c oxidase II and ATPase subunit 6, where it is normally located in other arthropods [16].

Since mtDNA sequences are commonly used in phylogenetic and population genetic analyses, we asked if our low-depth GSS strategy would typically recover a complete mt genome sequence in other species. Therefore, using the same methods as for *M. scalaris*, we assembled the 15.8 kb *D. pseudoobscura bogotana *mt genome from our GSS reads (Additional file 4). For *D. pseudoobscura*, 1.5% of the GSS reads were mitochondrial as compared to 0.5% for *M. scalaris*. The higher relative abundance of mt reads in *D. pseudoobscura *allowed for the assembly of the complete mt genome with a mean of 20× coverage. These results demonstrate that a relatively small number of sequences can recover complete mt sequences in GSS studies of some species.

### Scan for Microsatellites

GSS reads were scanned for the presence of all possible perfect di- or tri-nucleotide repeat motifs with the program Microscan.plx http://www.biology.duke.edu/noorlab/NoorSoft.html to find potentially variable microsatellite loci. Only 37 microsatellites with 10 or more repeat units were identified through this approach in *M. scalaris*. This was apparently not due to a failure of the program to detect microsatellites, as it recovered 429 such microsatellites when run with the *D. pseudoobscura bogotana *GSS sequences, despite there being 25% fewer sequence reads than in the *M. scalaris *GSS. The *M. scalaris *genome therefore appears to harbor a much smaller number of microsatellites. From these sequence reads, we were able to design primers and identify variability among strains at a subset of these microsatellites (data not shown).

### Identification of Gene Homologs

Homology searches were conducted to find *M. scalaris *reads with significant similarity to annotated genes and functional elements in other Dipteran genomes. Evaluating the number of "true" matches between *Drosophila melanogaster *gene sequences and *M. scalaris *GSS reads is challenging because of multigene families within both species, including histones, tRNAs and rRNAs. Hence, a single *D. melanogaster *gene may resemble many *M. scalaris *genes, and vice versa. Considering this issue, we examined the number of sequences with similarity between *D. melanogaster *and *M. scalaris *in two ways. As a first approach, we used BLAST to identify which genes in *D. melanogaster *bore significant similarity to the nonrepetitive *M. scalaris *GSS set. With an e-value cutoff of 10^-5^, we found 815 *D. melanogaster *coding genes with at least one significant match to a *M. scalaris read *(Additional file 5). However, this approach can still identify multiple similar *D. melanogaster *genes matching a single *M. scalaris *sequence trace. To address this, we then eliminated all redundant hits to single *M. scalaris *traces, and identified 330 unique *M. scalaris *reads with significant sequence homology to an annotated *D. melanogaster *gene.

For comparison, we also used the annotated genomes of *Drosophila virilis *and *Anopheles gambiae *to conduct gene homology searches. With *D. virilis*, 913 potentially homologous genes were identified, although most of the genes are uncharacterized in the *D. virilis *genome annotation (Additional file 6). As with *D. melanogaster*, we eliminated all redundant hits to a single *M. scalaris *sequence trace, which identified 306 unique *M. scalaris *reads with significant homology to a *D. virilis *gene. With *A. gambiae*, only 133 potential homologs from 98 unique *M. scalaris *reads were identified (Additional file 7), likely due to the combination of the smaller number of annotated genes in the *A. gambiae *genome and the longer divergence time between *M. scalaris *and *Anopheles *relative to *M. scalaris *and *Drosophila*. In all, 520 *M. scalaris *reads were identified as potential coding sequences by homology searches performed with other Dipteran genomes.

## Discussion

Although our preliminary sequencing of the *Megaselia scalaris *genome resulted in extremely low-coverage (between 0.05× and 0.10×), we were able to perform a number of bioinformatic analyses that provided useful information for characterizing this genome as well as generating various genomic resources. We were able to characterize numerous repetitive sequences in the genome, including some with homology to known elements and some that have not been characterized previously. Useful resources such as a nearly complete mitochondrial genome sequence and microsatellite markers were also easily developed from the GSS data. Moreover, partial sequences for hundreds of orthologs of *Drosophila *and *Anopheles *genes were generated.

An assumption laden in some of our analyses is that the genome survey sequences studied are "random" segments from across the genome. We cannot exclude the possibility that certain regions of the genome were more or less likely to be surveyed due to features such as GC-content. Indeed, we observed coverage of the mitochondrial genome was lower or missing in the most extremely A-T rich regions. This bias may have resulted from the sequencing process itself or issues with sample preparation and/or library generation. Nonetheless, the approaches here provide a first, albeit imperfect, approximation of various features of a previously unexplored genome, and several of our conclusions do not depend upon a truly random sampling of the genome.

*M. scalaris *was chosen for partial genome sequencing because of its interesting natural history and potential to become a model species in ecology and evolutionary biology. Previous work on *M. scalaris *has already revealed much about its ecology, development, sex-determination system, and life cycle [reviewed in [9]]. The species is widely distributed and many aspects of its ecology are peculiar. For example, *M. scalaris *larvae are notable for the wide range of organic matter on which they can feed; reportedly the widest range of any insect [9]. Because larvae are also facultative parasites, they can enter open wounds and therefore pose some threat to human health, especially in the developing world [17-19].

A complete *M. scalaris *genome sequence would also strengthen comparative and evolutionary genomic studies of the Dipterans. While there are completed genomes for 12 *Drosophila *species [20] and the mosquitoes *Anopheles gambiae *[21] and *Aedes aegypti *[22], no genome sequence is currently available for any Dipteran species outside of the *Drosophilids *and mosquitoes. A phorid fly such as *M. scalaris *would also serve as a good outgroup in comparative genomic studies of the Drosophilids. For example, the genome of *M. scalaris *could facilitate the identification of regulatory elements and assessing patterns of evolution, as has been recently suggested also for Tephritids [23].

### Applications of Low-coverage Genome Sequence

We anticipate that researchers studying a wide range of non-model taxa will be drawn to newer, less-expensive genome sequencing technologies, often for generating microsatellites [2,3] or other markers [24] to survey population variability and connectivity, phylogenetic position, and other questions. Based on our study of *M. scalaris*, using 454 pyrosequencing to sequence genomic DNA appears to be an effective strategy for generating low-coverage sequence data, with read-lengths amenable for assembly or BLAST [25] analyses. Sequence reads also appear to be distributed throughout the genome, allowing for partial coverage of many functional elements and hundreds of orthologs of known genes. Thus, low-depth sequencing provides mostly new sequence and avoids the high redundancy seen in large-scale genome projects.

The ability to find repetitive sequences is another important test of the applicability of survey sequencing since identifying and masking repetitive sequences can be crucial for accurately estimating genome coverage, identifying low-copy "gene space", and assembling large contigs. We identified over 100 *M. scalaris *transposable element copies by homology searches, most of which were LTR retroelements and non-LTR retrotransposons. These REs could be masked in future genomic work in *M, scalaris*, facilitating assembly of the short sequence reads obtained through 454 or other short-read sequencing. Low-coverage genome surveys therefore appear to be an effective way to identify repetitive sequences, as several previous studies have successfully identified repetitive sequences with low genome coverage in other systems [6,8,26,27].

While available programs like RepeatMasker (Smit and Green, unpublished data) and others can identify previously known REs, identifying novel REs in unassembled genomes remains problematic. Our REFinder.plx program was designed to quickly identify as many novel REs in unassembled genomes as possible. We further validated this program by applying it to comparable GSS from a species with a fully sequenced and assembled genome, *Drosophila pseudoobscura*, and identifying known elements. However, it was not designed to detect all classes of transposable elements and, because the program works by assembling and identifying potentially repetitive sequences in contigs, it can only identify REs in tandem arrays. It should also be noted that our program was not designed to identify higher-order repeats or identify the exact boundaries of REs. Other programs for *de novo *detection of REs, such as ReAS [28] or ReRep [29], may provide better detection of other classes of repeats, such as interspersed elements, in low-coverage genome surveys. It is also possible that some REs we detected are hybrids of different elements or that some non-repetitive flanking ends of REs were incorporated. Nonetheless, it provides a useful starting point for characterizing a novel genome of its repetitive element content.

Since no attempt to remove mtDNA from nuclear DNA was made prior to sequencing, mtDNA sequences were present in high copy number, which allowed us to assemble most of the *M. scalaris *mt genome. Even more encouraging was that we were able to assemble a complete mt genome at 20× coverage from the *D. pseudoobscura bogotana *GSS reads. This suggests that low-coverage genome surveys can also be an easy way of obtaining mtDNA sequences for phylogenetic studies and markers for population genetic studies. The proportion of mitochondrial traces was 0.5% (648/129,080) for the *M. scalaris *GSS and 1.3% (1299/98,451) for *D. p. bogotana*, consistent with the estimated greater nuclear genome size of the former (330-540 megabases vs. 185 megabases [30]).

While it would be helpful to know exactly how much sequence data is needed to completely cover a mt genome, this cannot be easily quantified. Based on a binomial distribution, the expected coverage of a target sequence given a certain depth of coverage or level of redundancy, R, can be approximated by the equation: **E**(Coverage) = 1 - e^-R^

Based on this relationship, for a 15 kb mt genome and a mean sequence read length of 200 bp, approximately 500 reads of mitochondrial sequence are needed to obtain full coverage. However, this approximation will not hold if sequence reads are nonrandomly distributed over the target sequence. For instance, a bias towards sequence reads being in G-C rich regions across the *M. scalaris *mt genome likely explains why we did not obtain the sequence of the A-T rich mitochondrial control region even though we recovered 648 mt sequence reads, far more than theory suggests are necessary. The amount of sequence required for full coverage of a mt genome therefore depends on biases in sequencing and DNA preparation, as well as biological differences among organisms (or even tissues) in mt copy number.

The point raised above for mt genome sequencing brings up a more general caveat for researchers using low-coverage GSS strategies. With low depths of coverage, the probability of obtaining complete coverage of any target sequence becomes exceedingly low. This holds true for coding sequences in the nuclear genome as well as organellar genomes. If specific sequences are the ultimate goal of genome sequencing, then more directed approaches would be more appropriate than our random GSS approach.

## Conclusion

We were able to generate genomic resources for *Megaselia scalaris *with very limited sequence data obtained through 454-pyrosequencing. Although this was a preliminary study, the data we have generated is both immediately useful and will be used to guide future larger-scale sequencing of the *M. scalaris *genome. We have also developed scripts for facilitating bioinformatics analysis of GSS data and made them available to the public. Our encouraging results suggest that low-coverage GSS approaches will become more popular among researchers working on non-model organisms, especially as the cost of next-generation sequencing continues to decline.

## Methods

### Sequencing and genome size estimation

We collected wild *Megaselia scalaris *individuals from populations in Durham, NC and inbred for 7 to 8 generations by crossing half-siblings. DNA for sequencing was prepared from adult males and females using a Puregene DNA isolation kit (Qiagen) and randomly sheared into fragments for sequencing. One-fourth of a standard 454 sequencing run was performed on a Roche GS-FLX sequencer at the Duke Institute for Genome Sciences and Policy. A low-coverage genome scan of *Drosophila pseudoobscura bogotana *was conducted in parallel with *M. scalaris *on the same 454 run (NCBI Short Read Archive accession SRA008268). Parallel sequencing of two different species allowed for the validation of sequencing methods and the computational analysis used in this study. The *M. scalaris *genome sequence traces were submitted to the NCBI Short Read Archive as accession SRA008342.

Estimates of genome size for both species were made using flow cytometry. We used the Partec^® ^UV Precise T kit for extraction and DNA staining of nuclear DNA, following the manufacturer's instructions. Adult *M. scalaris *males and females were run separately in the kit, both alone and with internal controls of *Drosophila pseudoobscura*. 2C values corresponding to the *M. scalaris *isolates were 4 times greater than those for *D. pseudoobscura*, suggesting a genome size roughly four times larger in *M. scalaris*. While this estimate is imperfect because of possible effects of differences in G-C content, it nonetheless provides a crude estimate. The genome size of *D. pseudoobscura *is 135 megabases [30], so that of *M. scalaris *would be roughly 540 megabases.

### Identification of Repetitive Elements *De Novo*

To identify a subset of highly divergent and lineage specific REs, we developed a Perl script (dubbed REFinder.plx) that can isolate tandem REs *de novo*. The Perl script is available at http://www.biology.duke.edu/noorlab/NoorSoft.html. Briefly, the program starts by building a contig around each sequence in a set of user-provided sequences. These contigs are constructed using an algorithm that uses BLASTn to find contiguous sequences and aligns them. Assembling reads into contigs allows the program to identify repetitive sequences that span the length of multiple reads. Each time the contig is extended, the program checks if the sequence being added to the contig aligns anywhere within the existing contig. If the new sequence aligns within the existing contig, the entire sequence between the new sequence and the matched sequence in the existing contig is extracted and considered a single potential repetitive motif. Reasoning that sequences present more than once in the GSS reads were more likely to be repetitive than sequences present in single copy, we only seeded the program with sequences present at least twice in the *M. scalaris *GSS.

### Mitochondrial Genome Assembly

We initially assembled the *M. scalaris and D. pseudoobscura bogotana *mitochondrial (mt) genomes by manually aligning contiguous but overlapping sequences, using the *D. melanogaster *mt genome as a scaffold. For *M. scalaris*, three gaps in the original assembly were filled by PCR amplifying fragments corresponding to the gaps. PCR fragments were then sequenced with Big Dye terminator sequencing reagents (Applied Biosystems) and run on an ABI 3730 sequencer. When multiple products were amplified, PCR products were first cloned using a TOPO TA cloning kit (Invitrogen) and then sequenced. After all gaps were filled, the original alignment was used as a reference to identify all mitochondrial sequences contained in the GSS reads. All mt sequences contained in the GSS were then aligned and assembled into a consensus sequence using the program SeqMan in the Lasergene 7.0 software package (DNAStar). Annotation of the mt genome was done with BLASTx for protein coding regions and BLASTn for non-coding regions.

### Scans for Microsatellites

The GSS reads were scanned for microsatellites with the custom-built Perl script Microscan.plx, which can identify arrays of di- and trinucleotide repeats and is specifically designed for use with FASTA-formatted genome sequence traces as input (for a review of other microsatellite search software, see [31]). The program is available at http://www.biology.duke.edu/noorlab/NoorSoft.html.

### Annotation of Functional and Repetitive Elements

To identify functional elements, we used BLASTn [25] to find *M. scalaris *reads with significant similarity to annotated features in other Dipteran genomes. Except where otherwise stated, all BLASTn searches were performed using the default settings and an e-value cutoff of 10^-5^. For comparisons with *D. melanogaster*, all annotated coding genes, including tRNAs and rRNAs, in release 5.13 from FlyBase [32] were queried against all *M. scalaris *reads. We repeated these searches using the annotated coding genes in release 1.2 of the *D. virilis *genome. For comparisons with *A. gambiae*, all protein-coding genes in the PEST AgamP3 assembly available from VectorBase [33] were used. To find previously characterized repetitive elements, we queried all REs present in the *Anopheles *and *Drosophila *libraries of release 13.11 of RepBase [34] against the *M. scalaris *reads.

## Authors' contributions

DAR executed virtually all of the research and analyses, and coded REFinder.plx. MAFN coordinated the project, performed the flow cytometry analyses, and coded Microscan.plx. Both authors wrote the manuscript.

## Supplementary Material

Additional file 1 ***M. scalaris *Repetitive Elements Identified Through Homology Searches**. List of all repetitive elements identified through homology searches of the *Anopheles *and *Drosophila *RepBase libraries. Click here for file

Additional file 2 ***M. scalaris *Repetitive Elements Identified Computationally**. Repetitive element motifs identified with REFinder.plx. The Seed ID is the GSS sequence trace around which the contig was built. The depth of coverage for was calculated as the average coverage over 40 bp windows. Click here for file

Additional file 3 ***M. scalaris *Mitochondrial Genome Consensus Sequence**. FASTA file containing the consensus sequence for our alignment of the *M. scalaris *mitochondrial genome with genes noted as Microsoft Word comments. Click here for file

Additional file 4 ***D. pseudoobscura bogotana *Mitochondrial Genome Consensus Sequence**. FASTA file containing the consensus sequence for our alignment of the *D. pseudoobscura bogotana *mitochondrial genome. Click here for file

Additional file 5 **Potential *M. scalaris *Homologs of *Drosophila melanogaster *Genes**. List of all *D. melanogaster *genes that showed significant sequence similarity to at least one *M. scalaris *GSS sequence trace. Click here for file

Additional file 6 **Potential *M. scalaris *Homologs of *Drosophila virilis *Genes**. List of all *D. virilis *genes that showed significant sequence similarity to at least one *M. scalaris *GSS sequence trace. Click here for file

Additional file 7 **Potential *M. scalaris *Homologs of *Anopheles gambiae *Genes**. List of all *A. gambiae *genes that showed significant sequence similarity to at least one *M. scalaris *GSS sequence trace. Click here for file
