# Supplementary material for: What can you do with 0.1× genome coverage? A case study based on a genome survey of the scuttle fly Megaselia scalaris (Phoridae)
Source: BMC Genomics. 2009 Aug 18;10:382. doi: 10.1186/1471-2164-10-382 (PMC2735751; doi:10.1186/1471-2164-10-382)
Supplement: Additional file 3 — M. scalaris Mitochondrial Genome Consensus Sequence. FASTA file containing the consensus sequence for our alignment of the M. scalaris mitochondrial genome with genes noted as Microsoft Word comments. [file 1471-2164-10-382-S3.doc]

>Megaselia scalaris - Mitochondrial Genome - April 14th, 2009

TAATTTTTTAAATCtATATAATAAATACAGAATATTACACAATTTTGTtACAAATAAATCAATTTTCATGTTTTTTTTTACTATAATGAATTGCCTGAAAAAAGGGTTAACCTTGATAGGGTAAATCATATAATTAAACATTATATTCATTATATTTAATAGAATTAAACTATTTCTAAAAGTATCAAAAACTTTTGTGCATCATACACCAAAATATAAAAAGATAAGCTAACTAAGCTATTGGGTTCATACCCCACTTATAAAGGTCATAATCCTTTTCTTTTTAATTTTtAAAAATTCTTATAAATTATTATTTGTTATAACATTAATGTTAGGAACTTTAATCACAGTCTCATCTAATTCATGATTAAGAGCTTGAATAGGACTTGAAATTAATTTATTATCATTTATCCCCCTTATAAATGATACAAAAAATTTAATATCATCTGAATCTTCTCTAAAATATTTTTtAATTCAAGCTTTAGCTTCATCTATTTTATTATTTTTAATGATTTATTATTTAATTAATATAAATCATGATTTTCTTACTTCCACTAATATTAATTTAGGAATAATATCTTCTTTAATATTAAAGAGAGGTATGGCTCCATTTCATTTTTGATTTCCTAATGTAATTGAAGGATTAAATTGAATTAATTCATTTATTTTATTAACGTGGCAAAAAATTGCCCCTTTAATATTAATATCTTATTTAATAACTAATTATTTATTAATCCCAATTATTTTATCAATAATTGTAGGTTCTTTAGGAGGATTAAATCAAACATCCTTaCGAAAAATTATAGCCTATTCTTCAATTAATCATTTAGGATGAATGGGGGCAGCAATAATTTATAGAGATAATTTATGATTAAATTATTTTTTATTTTACTCATTTCTATCAGTTACTCTAATTTATTTTTTCAATATTAATAAATTAtTTTTTATAAACCAAATATACTCAATATTCAACTATTCAAATGAAATAAAATTAATTTTATTTCTTAATTTTTTATCATTAGGAGGACTACCTCCATTTTTAGGATTTTTCCCAAAATGAATAGTTATTCAAATAATATCAAGTAATCAATTATTTTTAATTACTATTATAGTAATATTAACTTTAATTACTTTATATTATTACATTCGAAttATGTTATGCAGGCTTTATAATAAATTATTATGAAAaTAAATGAAATTCTTTAAATATTATaAAATTAAATAATATAACAATTTATTTAATTTTATCATTTATTTCAATTTTTGGGTTAATAATTATTAATCTCTTAtTTTTTtATTTTTTAAAGGCTTTAAGTTAAAATAAACTAATAGCCTTCAAAGCTATAAATATAAGATAATCTTTTAAGCCTTAATAATTTTATAATTATTCCTTTAGAATTGCAGTCTAATATCATTATTGACTATAAAGCTTGATTAAAGAAAATAAATTTTCGTACATAGATTTACAGTCTATTGCCTAAACCTCAGCCATTTAATCTTATTGCAACAATGATTATTTTCAACTAACCATAAAGATATTGGAACTTTATATTTTATTTTTGGAGCCTGAGCTGGAATAGTAGGAACATCTTTAAGTATTATAATTCGAGCTGAATTAGGGCACCCTGGTGCTTTAATTGGTGATGATCAAATTTATAATGTAATTGTTACTGCCCATGCATTTATTATtAATTTTTTTATAGTAATACCTATTATAATAGGAGGATTTGGAAATTGATTAGTTCCCCTAATATTAGGGGCACCTGATATGGCCTTTCCACGAATAAATAATATAAGTTTTtGAATACTTcCCCCTTCTCTAACTCTTTTATTAGCAAGAAGTATAGTAGAAAATGGAGCCGGAACTGGTTGAACAGTTTATCCGCCCCTATCTTCTAGAATTGCCCATAGAGGAGCTTCAGTCGATTTAGCAATTTTTTCATTACATCTTGCCGGAATTTCTTCTATTCTTGGAGCAGTAAATTTTATTACTACAATTATTAATATACGATCTACAGGAATTACTTTTGATCGAATACCTTTATTTGTATGATCAGTAGGTATTACTGCTCTTTTATTATTACTTTCACTACCTGTTCTAGCAGGTGCTATTACTATACTATTAACAGATCGAAATTTTAATACATCATTCTTTGATCCTGCGGGAGGGGGAGATCCAATTCTATATCAACATCTATTTTGATTTTTTGGACACCCTGAAGTTTATATTTTAATTTTACCTGGATTTGGTATAATTTCTCATATTATTAGTCAAGAATGTGGTAAAAAGGAAACCTTCGGTTCTCTTGGAATAATTTATGCTATATTAGCTATTGGTCTTCTTGGATTCATTGTATGAGCTCATCATATATTTACTGTAGGAATAGACGTTGATACACGAGCTTATTTTACGTCTGCAACAATAATTATTGCTGTACCTACAGGAAtttAAAATTTTTAGTTGACTAGCTACACTTCATGGTACACAATTAAATTATTCACCTGCTCTACTATGAGCCTTAGGATTTGTTTTCTTATTCACAATTGGGGGATTAACAGGAATTGTTCTCTCTAATTCATCTATTGATATTGTATTACACGATACATATTATGTAGTAGCTCATTTTCATTATGTACTTTCTATAGGAGCTGTATTTGCTATTATAGCAGGTATTATTCACTGATATCCTTTATTTACTGGTTTAACCCTTAATAATAATTTATTAAAGACACAATTTACTACTATATTTATTGGAGTAAATTTAACTTTTTTCCCTCAACACTTCTTAGGATTAGCCGGTATACCACGACGATACTCTGATTATCCTGACGCTTATACTACATGAAATATTATTTCATCAATTGGTTCTTACATTTCATTTTTTAGTATTATTTTATTTTTATATATTATTTGAGAAAGTTTTATAGCTCAACGTCATTTATTAAATCCTtCCAATATAAATTCATCAATTGAGTGATATCAAAATACTCCTCCTGCAGAACATAGCTATTCTGAATTACCTTTATTAACTAATTAATTCTAATATGGCAGATTAGTGCAATGGATTTAAGCTCCATATATAAAGTATTTTACTTTTATTAGAAACAAATGTCAACATGAGCTAATTTAAATCTTCAAGATAGAGCTTCCCCATTAATAGAACAATTAACtATTTTTTCATGACCATGCaTtATTAATTTTAGTAATAATTACTGTTTTAGTATCTTATATAATAGTAACTTTATTTTTTAAtTCAATTTACAAACCGATATCTTTTACATGGTCAAACTATTGAAATTATTTGAACTATTTTACCTGCTATCACTCTCTTATTTATTGCTTTCCCATCTCTTCGATTATTATATTTAATTGATGAAATTAATGAACCAATAATTACATTAAAGTCAATTGGGCATCAATGATACTGAAGCTACGAATATTCAGATTTCATAAATGTAGAATTTGACTCTTATATAATTCCTACTCATGAATTAGAAAATAATGGCTTCCGATTATTAGATGTAGATAACCGAGTTGTTTTACCTATAAATTCTCAAATTCGTGTATTAGTAACCGCTACTGATGTAATTCACTCATGAGCCATTCCAGCTCTTGGTGTAAAAATTGACGGAACTCCCGGACGATTAAATCAATCTAATTTTATAATTAATCGACCCGGATTATTTTTCGGTCAATGTTCTGAAATTTGTGGGGCTAACCATAGTTTTATACCTATTGTAATTGAAaGTATCCCAATAAATTATTTTATGAAATGAATTTCTAATAATATTTAATTCATAAGATGACTGAAAGCAAGTACTGGTCTCTTAAACCATTTTATAGTAGAATAGCACCTACTTCTTAaTRAAGAAAAAAAAAAAAAAAAAAAAAAAAAAANNNNNNNNNNNNNNNNNNNNNNNNNNNNNNNNNNNNNNNNNNNNNNNNNNNNNNNNNNNNNNNNNNNNNNNNNNNNNNNNNNNNNNNNNNNNNNNNNNNNNNNNNNNNNNNNNNNNNNNNNNNNNNNNNNNNNNNNNNNNNNNNNNNNNNNNNNNNNNNNNNNNNNNNNNNNNNNNNNNNNNNNNNNNNNNNNNNNNNNNNNNNNNNNNNNNNNNNNNNNNNNNNNNNNNNNNNNNNNNNNNNNNNNNNNNNNNNNNNNNNNNNNNNNNNNNNNNNNNNNNNNNNNNNNNNNNNNNNNNNNAAAAAAAAAAAAAAAAAAAAAAAAAAATWAWWAATWATAAATWATAAATTATAAATTGTGTATGATTTGGGTTTTGAGATAAAATAAATCAAAATATTTATTTTAAAAAAATAGTTTAAAGTCAAAATAATAGATAAAAAATTATTTAAAATAGTTACATTACTCAATTATAAAAAATTAGTTAAATTATAACATTAGTATGTCAAACTAAAATTATTAAATTATTAATATTTTTTGATTCCACAAATAGCCCCAATTAGTTGATTATTTCTATTTTTAATCTTTTCTATTACTTTTATTATATTTAATATCTTAAATTACTACATTTTTCTCTACTCCCCAATTTCTRAGGAAAAAAGAGAAAAAAAAAGTCTTAAATCATTAAACTGAAAATGATAACAAATTTATTTTCTGTATTTGACCCTTCTTCAAGTATCTTAAATCTATCATTAAATTGATTAAGAACATTTCTTGGATTATTAATAATTCCTTCAATATTCTGATTAATTCCTTCTCGATACCATGTAATTTATAGTAATATTTTATTGACTTTACATAAAGAATTTAAAACATTATTAGGACCTGCTGGACATAATGGAAGAACTTTTATTTTTATCTCTCTATTTTCATTTATTTTATTTAATAATTTTATAGGATTATTCCCTTATATTTTTACTAGAACAAGTCACTTAACTTTAACTCTTACTTTAGCTTTACCTCTATGATTGAGCTTTATGTTATACGGGTGAATTAATCATACACAACACATATTCGCTCATTTAGTTCCTCAGGGAACCCCGGCTATTTTAATACCTTTTATGGTTTGTATTGAAACTATTAGTAATGTAATTCGACCAGGAACATTAGCTGTTCGATTAACTGCTAATATAATTGCAGGACATCTTCTAATAACATTACTAGGAAATACAGGGCCTTCAATATCTTTAATTTTAGTGAATATTCTTATTATTACTCAAATTGCCTTATTAGTTCTTGAATCAGCTGTATCAATTATTCAATCTTATGTATTTGCAGTTTTAAGAACTTTATATTCTAGAGAAGTAAACTAATGTCAACACATTCAAATCACCCATTCCATTTAGTTGATTATAGCCCTTGACCTCTAACTGGGGCTATCGGAGCTATAACAACTGTTTCAGGTATAGTAAAATGATTTCATCAATATGACTCATCATTATTTTTAtTAGGAAATATTATTACGATTTTAACCGTTTATCAATGATGACGAGATGTATCACGAGAAGGGACTTTTCAAGGACTTCACACATATATAGTAACTATTGGATTACGATGAGGAATAATTTTATTTATTATTTCTGAAGTATTTTTCTTCATTAGTTTTTTTTGAGCTTTTTTTCATAGCAGCCTTTCCCCAACTATTGAACTAGGAGCTATATGACCTCCTATAGGAATTCAAACTTTTAATCCATTCCAAATTCCTTTATTAAATACTACTATTCTTCTATCATCTGGAATTACAGTAACTTGAGCACATCATGCATTAATAGAAGGTAATCATTCACAAGCTACTCAAGCTTTATTTTTCACAGTAATTTTAGGTATTTACTTTACAATTTTACAAGGGTACGAATATATTGAAGCCCCATTTACAATTGCTGATTCTGTTTATGGCTCAACTTTTTTtATGGCCACAGGATTTCATGGAATTCATGTATTAATTGGAACAACATTTTTATTAATTTGTTAATTCGACATATAAATTTTCATTTCTCTAAATCTCATCATTTTGGATTCGAAGCTGCTGCATGATACTGACATTTTGTAGATGTAGTATGATTATTCCTTTACATTACTATTTACTGATGAGGAGGATAAATTATTTATATAGTATAAAAGTATATTTGACTTCCAATCAAAAGGTCTATAAAATTATAGTATAAATAATTTTAATAATATTAACTATTGGTTTTATTCTTCTATTACTTGCTTGTATCATAATTATTTTAGCTACAATTCTTTCTAAAAAAaCTATTTTAGACCGAGAAAAATGTTCTCCTTTTGAATGTGGTTTTGATCCAAAGTCATCTTCCCGTCTACCATTTTCCCTACAtTTTTTTTTAATTGCGATTATTTTTTTAATTTTTGATGTAGAAATCGCTCTTATTTTACCTATAATTTTAGTATTAAATTTTTCTAATATTACTGTATGATTATTAACTAGCGCGTTCTTTATTTTTATTTTATTACTTGGATTGTACCATGAATGAAACCAAGGAGCTTTAAATTGAACTAACTAAGGGTTGTAGTTAATTATAACATTTGATTTGCATTCAGAAAGTATTGATTTATCAATCTACCTTAGAATATGAAGCGATCCATTGCAATTAGTTTCGACCTAATCTTAGATAAATTTTATCCTTATTCTTTAATTGAAGCCAAAAAGAGGCTTATCATTGTTAATGATATCATTGAAGAATAACTTCCAATTAAAGAAATATGATGCCCAAaGAAAAAGCTGCtTAACTTTTTACTTTAATGGTTAAATTCCATTTATATTTCGATTTATATAGTTTAACAAAAACATTACATTTTCATTGTAAAAATAAAAAATTAtTTTTTATAAATTACTAAAATTAATTATTAATATTCAAAGATTAAATAAATCTCCCTAACATCTTCAGTGTCATACTCTAGTTATAAGCTATTTGAATAAATTATAATTATTATAATGATAATAAATATTCATAAAACAAATACTCTCATATAAATCTTAAAATTATTATTTTGAACAAATTGATTATATTGAGATATATTAATAAATATTTTAAATATTATTTGGCCCCCTAAATACTCTGATCAACCTTGGTCTATTGACTTATAAATTATTTTACCAAAATTTAAAGGATAATTAATAATTCCGTAAGTTGAAATATAAGGTATAAATCATATTGATCCTAAAAAATAAGAAATATTATATAAGTCTAAAGATTTATTATAAAAGTATAAATTAACCAATGAAATGAAATAACCAAATAAACCCCCAGTAATACAAACAAATAAAGTCAACTGTTTTAAATAAATAGGTAAACAAATCATATAAGGAGTAGGAAAAATTAATCAATTTAAAATTCTACCTCCAATAATTGATATAATAAGTAAACCTATTATACCCTTTAATATTACTCATCCTTCATCTCTTAAAACATTTAATCTTCTACAATTTAAATCTCCAGTAACTGAATAATATACAAGTCGAAAAGAATAACAAACAGTTAATCCGGTaAGAAAAAAAGAATAAAAAGAAAATTAACATATTTACATTACTTATAGATACAATTTCTAAAATTATATCCTTTGAATAAAATCCGGCTAAAAAAGGTATTCCACATAATGCTAAATTAGAAATATTAAAACAAGCAGTAGTAATAGGCATATAAATTCCTAAACCCCCTATAACACGAATATCTTGAAAATTATTTATGTTATGAATAATACTTCCCGCACATATAAATAATAAGGCCTTAAATAAAGCATGAGTTAAAAGATGAAAAAATGCTAATTTATAGTACCCTATAGATAAAATTCTTATTATTAACCCTAACTGAGAAAGTGTAGACAAAGCAATAATTTTCTTTAAATCAAATTCAAAGTTAGCGCCAAGGCCTGCTATAAACATTGTTAATCCTGAAATTAACAATAAAATTTTTCCAATTATTGAATCCACTAACAAAAAATTAAATCGAATTAATAAATAAACACCCGCTGTTACTAAAGTTGATGAATGGACAAGAGCAGAAACAGGAGTAGGAGCGGCCATAGCCGCTGGAAGTCAAGAAGAAAAAGGAATTTGAGCTCTTTTTGTTATAGCGGCTAAAACAACTAAAATCCCCACAATTTGTATTTCTCTAGTTATTTTTATAAATTCTAAATAAAAAATATAATTTCAACTCCCAAAATTCAATATTCAAGCAATAGCTAGTAATAAAGCTACATCTCCAATTCGATTAGATAGAGCAGTTAATATACCAGCACTATAAGACTTTACATTTTGAAAATAAATTACTAAACAATAAGATACTAAGCCTAACCCATCTCACCCTAATAAAATACTAATTAAATTAGGTCTGATAATTAGTAATATTATAGAACTAACAAATATTAAAACTAATATAATAAATCGATTAATATTTTCATCTTCACTCATATATTCCTTTCTGTAAAAAATTACTAAAGAAGAAATTAATAAAACAAATGATATAAAAATTAAACTTATTCAATCTAATAAAATTGTTATAACAATTGATAAGGAATTTAAACTAACAATTTCTCATTCAATAAATAATCTATAATCTTGAGATAAAAAATTTACCCCYTCTCaACAAAACAAGTTAAACTACAACTAATTAAACAAAAAAaTaCTAATTAAACAAATAGAAATATATTTCACGATTTAAAATGAATTAATTCATATCTTTGACACCACAAATCAAAATTTTtAATAAACTATTTAAATATAAAAaTATTAAAAATATATCACTTTTTATAATTAATAAATTTAAAGGAAATCAATGAAGAAACAATAATAAATACTCTCGTCTTAACCCATTACTAAAACAATAAATACCTCTATACAATTGTCCATGTTGAGTATAAGAATATAAATATAAAGTATAAGCAGCTCTAAAAAAAGAAATTAAAGCAATTATAATTATTGAAACTCAAGATCAACAAACAATAGAATTTAATAAACTAATTTCGCTTAATAAATTAATAGTAGGAGGGGCAGCCATATTAGCAGAACTTAACAAAAATCACCATAATGCTATTCTAGGCATAAAATTTAAAAATCCCTTATTAATTAATATGCTTCGTCTACCTCTTCGTTCATATATAATATTAGCTAAACAAAATAACCCTGAGGAACATAATCCATGAGCAATTATTAATCCATATGAACCATAATAACCTCAATATAATAAAGTTATTAACCCACTTACCACAATTCCTATGTGAGCAACAGAAGAATAAGCAATCAATACCTTTAAATCAGTTTGTCGTAAACATATTAGACTCACTAAAAAGCCCCCCACTAAACTAATAGAAATTCAAACAAAATTATATTTTAATCCTAGTTCCTGAATTAAAATTAATGAACGCAATAAACCGTATCCTCCTAATTTAAGTAATACTCCCGCTAAAATTATTGACCCAGAAACAGGGGCTTCAACATGAGCCTTAGGCAATCATAAATGAACCATAAATATTGGTATTTTAACTAAAAAGGCTATAATTAAACCAAAATACAGTATTTCATAATTATAAATTTaCTTCAAGTATATAGAAATTTAAAGTATTTAAATTTTTATATAAATAAAAAATACTTACTAATAAAGGCAATGAAGCTAAAAGAGTATAAAATAATAAATATACTCCAGCTTGTAAACGTTCAGGTTGATATCCTCACCCTAAAATTAATAATAATGTGGGAATAAGGCTTCTTTCAAAAAATACATAAAATAAAAATAAATTTACAGTTCTAAAACTTAAAAATAAAAAAATATTAATATAACAACATTAATTAAAAATAAAtTTTTATAATTATTTATAAAATTTACTAAACCACTTGATATAATTATTAAACTACAAATCCACAAACTTAATAAAATTATCCCATAAGAAAATAAATCACAACCTAAAAwGTATCTTAAATTTATTCAATAATTATTAAAATAATTATTAATAATAAATAAAAAACTTACTAAAAATAACATATTTTGAACCATTCAAAATATCCGCTTAAATAAACATAAAGGTGTCAATATTAAAATAAAAAACAAAAATTTTARCATATTAAAATATTAAAGGATTGAAAATAATTATTCCCATGCGTACGAATTATAGAAACAAGAATTGATAGCCCTAATGCCCCTTCACATACTATAAAAATTAAAAATATTATTAATACATATATTTCACTATTATAATTTACTAAAAATAAAATAAAAATATAAATAAACTCAACATAATGAATTCTAAACTTAACAACATACTTAATAAATGCTTACGCAAAGATACAAATACATATACTCCTCTCAAAAATAAAACAATTACCAATATATTATATATTATCATTAGTTTTAATAGTTTAACAAAAACATTGGTCTTGTAAATCAAAAATAAGATTATTATTCTTTTAAAACTTCAAGAAAAAAGACACTTCTTTATCATTAATCTCCAAAATTAATATTTTAAATAAACTATTTCTTGATATTATACAAAATTTATTATTTTTTTTATTTTATTTACTGCTATTTTATTTATTCAATTAAATCATCCTCTAGCAATAGGATTGACTTTATTAATTCAAACACTATTTATTTGTCTATTAACAAGCTTAATAAATGAAACATATTGATTCTCCTATATTTTATTTTTAGTATTCATAGGAGGATTATTAGTTTTATTCATCTATGTAACATCTTTAGCCTCAAATGAAACTTTTTCTCTATCTTTCAATATCGTTTTTGCGGCAATAATTTTATTTTCAATCTCTATATTAATTATATATTTCTACGACTCTATAAATACGAATAATTTTTTTTATTCAAATAATATATTACCTGaAAATACAATCAATTTAATTAAATTATTTAATTACCCAACAAATTTAATTACAATCCTATTAATAAATTATTTATTAATTACTTTAATTGCTGTAGTTAAAATTACAAATATTTTTTATGGCCCATTACGTCATATATATAATTAATGAACAAACCATTACGATCTTCACACCCTTTATTTAAAATTGCTAATAATGCATTAGTAGACTTACCTGCTCCTATCAATATTTCTTCCTGATGAAATTTTGGTTCTTTATTAGGTTTATGTTTAATTATTCAAATTTTAACTGGATTATTCCTTGCAATACATTATACAGCTGATATTAATTTAGCTTTTAATAGAGTAAATCATATTTGTCGAGATGTAAATTATGGATGATTTTTACGAACTTTACATGCTAATGGTGCATCATTTTTCTTTATTTGTATTTATATGCATGTTGGCCGAGGAATTTATTATAACTCTTATTTATATATTCCTACTTGATCAGTGGGAGTAATTATTTTATTCTTAGTAATAGGAACTGCTTTTATAGGATATGTTCTTCCTTGAGGGCAAATATCTTTTTGAGGAGCTACTGTAATTACAAATTTATTATCAGCTATTCCTTATTTAGGGACTGATTTAGTACAATGATTATGAGGAGGATTCGCTGTCGATAACGCTACATTAACACGATTTTTCACATTTCATTTTATTTTTCCTTTTATTGTATTAGCTCTTACATTAATTCATCTTCTTTTTTTACATCAAACTGGATCAAATAATCCCACAGGATTAAACTCAAATTCAGATAAAATCCCCTTTCACCCATATTTTACTTATAGGGATATTGTTGGATTTTTAATTTTATTAATAACTTTAACTTTATTAACTTTAGTTAATCCTTATTTATTAGGAGACCCCGATAATTTTATCCCAGCAAATCCTTTAGTAACACCTGCCCATATTCAACCTGAATGATATTTTCTATTTGCATATGCTATCCTTCGGTCAATTCCTAATAAATTAGGAGGAGTAATCGCCCTTGTACTTTCTATTGCCATTTTATTAATTTTACCCTTCTCTCATACAAGTAAATTTCGAGGTCTACAATTTTACCCTATTAATAAAATTTTATTTTGAATTATAGTAATTACAGTAATTTTATTAACTTGAATTGGAGCTCGACCTGTAGAAGACCCATATGTCTTAATCGGACAAATTTTAACTGTAGTTTATTTTTCTTATTATTTAATTAATCCTTTAGTTTCTAAATGATGAGATAATCTATTAATTTGGTTAATGAGCTTGAATAAGCATATGTTTTGAAAACATAAGATAGAATTTTATTTTCTATTAACTTTACTAATTTAAATTAATAATATGAATATTAATATAAAAaTTTTAAATCCTATAAAAAACATTAAATAATTTAAAGAAAAAGGTAAAAAAGACTTTCAAGCTAAATATATTAATTTATCATATCGAAATCGAGGTAAAGTACCTCGCACTCAAAATAAAAACAAAAGATATAAATATTAACTTAAAATAAAAATAAAAAAGAAGTTATATCmTaCCCCCTAAAAATATTAATACGAATAATATTCTyATAAATAAAATTCTCGCATATTCAGCTAAAAAAATwCAATGCAAAyCCCCCTCTTCTATATTCAATATTAAATCCAGACACTAATTCCGATTCTCCTTCTGCAAAATCAAAAGGAGTACGATTAGTCTCAGCTAAACAAGAGCTAAATCAAACTAACCCTATGGGCAATATTATAAATAGAAATCAAATATATGTTTGATAAAATATAAATTGTATTATATTATAATTCCCAAaTTAAAAAAACAAAAGATAAaTAAAATTAAAGCTAATCTAACTTCATATGAAATAGTTTGAGCAACAGCCCGTAAACTTCCTAATAAAGCATAATTAGAATTTGAAGATCATCCAGCAATTATAACAGTGTAAACCCCTAAACTTGTACAACATAAAAAAAACAACAAACCTAAATTAAATCTATATAAATTAATTAACATAGGTATACATATTCAAGTTATTAAAGCCAAAAAAACGAAAAAACAGGAGATAAATAATAAGGTAGAAAATTAGACACTAAAGGATAAGTTTGTTCTTTTGTaAAaTAACTTAATTGCATCACAAAAAGGTTGAGGAATTCCTATAATCCCTACCTTATTAGGTCCCTTACGAATTTGAATATATCCTAAAACCTTTCGTTCTAACAATGTCAAAAAAGCAACACTTACTAATACACAAATAACAAGTAATACACTTCCAATTAATATTAAAACTACATCTATATAAAACAAGTACTATTTATAGAATAATCTATATACATAAATTCTAAATTTATTGCACTAATCTGCCAAAATAG..TATATTAATAAAATTCAATATATAAAATCTATAATTTATATATTTGGTCCTTTCGTACTAAAATATATtAAATTTTTAAAGATAGAAACCAACCTGGCTTACGCCGGTCTGAACTCAGATCATGTAAGAATTTAAAAGTCGAACAGACTTTAAGTTTAAGCTGCTGCACCTAAAACTGTATCTTAATCCAACATCGAGGTCGCAATtCTTTTTTATCAATATGAACTCTCTAAAAAATTaCGCTGTTATCCCTAAAGTAACTTGATTTCTTAATCATTAATAATGGATCAATAATTCATTAATTTATGTTTATTATAAATTAAAAGTTTAACAAATTTTAATATCACCCCAATAAAATATATAAATTTATAATAATTTAATTTATCTATATAATTAAAATAACTTATATATAAAGATTTATAGGGTCTTCTCGTCTTTTAAAATTATTTTtAGCTTTTTAACTAAAAAATAAAATTCTATTATAAATTTATATGAAACAGTTAATATTTCATCCAACCaTtCAtACCAGCCTTCAATTAAAAGAmTAWyTGyAKWATKCKMCCTTTTGCACAGTTAAGATACTGCGGCCATTTWAAAAATTTCAGTGGGCAGGCTAGACTTTAAATTAAATTCAAAAAGACATGTTTTTGTTAAACAGGTGAATATTAATTTTGCCGAATTCTTTATATAAACCTTTCAAATAATTTTTATTTATAAAATTAAATATACTAATTATATCATTATTTCTTTATTTTTTAAATTAAAATAAATATTTTCATAAAAATTTAAAATATATTAAATCATATTATACATAAAATAAATTATAACATAATTATTAATAATTGCTAGTTATAAGCATATATTTATTAAATTAATTTAATAATTTATAAAAATTTATTAAATAGCTTATCCCATTTAATAGTAAAATTTATAATTAATTTGATTAATAATTAAATTAATTATTATAAATTTCTAAATTAAATTTATTTCTGAAAAAACTAGATACCATTAAAAACGAATAACATTTCATTTCTAATAAATTATTAAAAATAGTTTTACTACAATAACTTTACTAATTTATTAACTCTTTTAAAATCGAGAAAATTATCAATAAATAACTTTAATTTGATACACCCTGATACAAAAGGTACAATAAATTAAATTTTCTTTTTTTATAAAAATTTTTCAAAATATTTCAATTTTCTTTCACAATACTAATACTCTATAATTTTTATTATTATTTTATTATAAATTACTAAAACATAAAATTTTATAATTATTTTTAATATAATTATTTAAAATTAAAAAAATTAATAAATAAAATTTAATCAATTTATAATGATTTGCACATAAATCTTTTCAATGTAAATGAAATGCTTTACTAATTAAGCTTTAAATTGT..ATTCTAGGTACACTTTCCAGTACACCTACTATGTTACGACTTATCTTATCTTAATAATAAGAGTGACGGGCGATATGTACATATTTTAGAGCTAAAATCAAATTATCAATCTTTATAATTTTACTATCAAATCCACCTTCAATAAATTTTTCATATTTATATCCGTATAAATAAATTTATTGTAACCCATTAAAAACTTAACTATAAACTGCACCTTGATCTGATATACAATTTTATTTAATTTATTGAAAATTATTATTCTTATAAAATTTTCTATAACGACGGTATACAAATTAATATATAAAATTAAGTAAGGTACATCGTGGAATATCGATTACAAAACAGGTTCCTCTGAACAGACTAAAATACCGCCtAAATTTTTTAAGTTTAAAGAATATAACTATTAATACTCAAGTTTTAAAATTACATTTTAAATAATAGGGTATCTAATCCTAGTTTATATCTAAAAtTTtCTAAGCTTCAACTATTCTAATAAAAATATTATAATTATAAAATTTCACCTAATATAATTAAtTTTAATATTATTTTAAATTTCGTTTAACTCAAAACTAAAAATATTTATTTGCATCTTTTGTCTAACCGCGACTGCTGGCACAAATTTAGTCAATACTATTTAATATTACTATTTCTAAATTTCTTTAATTAATAAAATTATTTACTACAATTATATAAAaTTAATATATTATTAAAATAAATTAAAATTCACGCAAAAACTTGTATATAAATTATATTAAAAATAAATATTA
